# Corrigendum to “Examining the relationship between nutritional status and wound healing in head and neck cancer treatment: A focus on malnutrition and nutrient deficiencies”

**DOI:** 10.1111/iwj.14876

**Published:** 2024-04-23

Chen Y, Li Y, Ceng Y, Li C, Li Y, Wang Y, Wang K. Examining the relationship between nutritional status and wound healing in head and neck cancer treatment: a focus on malnutrition and nutrient deficiencies. *Int Wound J*. 2024;21(3):e14810. doi: 10.1111/iwj.14810

The third author's name was incorrectly spelt as Yaqi ceng. It should have been Yaqi Zeng.

We apologize for this error.

